# Inclusion of child-relevant data in the development and validation of heat vulnerability indices: a commentary

**DOI:** 10.1088/2752-5309/acdd8a

**Published:** 2023-06-22

**Authors:** Kate R Weinberger, Blean Girma, Jane E Clougherty, Perry E Sheffield

**Affiliations:** 1 School of Population and Public Health, University of British Columbia, Vancouver, BC V6K0G8, Canada; 2 Department of Environmental Medicine and Public Health, Icahn School of Medicine at Mount Sinai, 1 Gustave L. Levy Place, New York, NY 10029, United States of America; 3 Department of Environmental and Occupational Health, Dornsife School of Public Health, Drexel University, 3215 Market Street, Philadelphia, PA 19104, United States of America

**Keywords:** extreme heat, temperature, children, morbidity, mortality, Heat Vulnerability Index

## 1. Introduction

The health impacts of extreme heat have been repeatedly linked to increased rates of illness and death. Research to date has documented a number of subpopulations at heightened risk during periods of extreme heat. While there is a large body of research linking heat to adverse health outcomes among adults, an emerging literature has also identified infants and young children as a heat-vulnerable population. Thus, there is an opportunity to further improve strategies and tools developed to prevent heat-related illness and death through consideration of this population. In this commentary, we examine the extent to which data that capture the vulnerability of children is incorporated into the development and validation of a specific tool: the Heat Vulnerability Index (HVI), a tool used to map spatial patterns of heat vulnerability within urban areas. Additionally, we make recommendations for how HVIs might be expanded or targeted to capture the impact of heat on younger populations.

## 2. Extreme heat and child health

Exposure to high ambient temperature is a well-established threat to human health. A large body of epidemiologic literature has documented associations between days of hot weather and morbidity and mortality due to a wide range of adverse health outcomes, including conditions directly due to heat exposure (e.g. heat stroke) as well as the exacerbation of other underlying chronic conditions (e.g. cardiovascular disease) [1, 2]. Recent estimates suggest that heat may contribute to as many as 12 000 deaths per year in the United States alone [3]. As the climate warms, this threat may be magnified as populations in the United States and around the world experience more frequent hot weather [4].

A major line of inquiry within the broader umbrella of heat-health research is the identification of subpopulations that are at heightened risk of experiencing illness or death during periods of hot weather. To date, groups identified as more likely to experience adverse health outcomes during hot weather include older adults, outdoor workers, people of a race other than white, people who live alone, people with lower income, people with certain chronic health conditions, people without access to air conditioning, and people who live in neighborhoods with less green space or more impervious surface cover [5]. The factors contributing to such heightened risk, often conceptualized as ‘heat vulnerability’, include not only physiologic sensitivity to heat, but also differences in the extent to which individuals are exposed to heat and the degree to which individuals or the communities where they live have the capacity to respond to heat (i.e. adaptive capacity) [6].

While much of the research on heat and health to date has focused on adults, infants and young children have also been identified as a population that is vulnerable to hot weather. Studies conducted among this age group have documented associations between heat and a wide range of health outcomes, including heat-related illness (e.g. heat exhaustion, heat stroke), dehydration, electrolyte imbalance, injury, and specific forms of respiratory disease (e.g. asthma, wheeze) [7]. Differences in thermoregulatory capacity and activity patterns compared with adults, as well as the dependence of very young children on adult caregivers for protection from heat (i.e. lesser ability to regulate their own heat exposure) may all contribute to the vulnerability of this age group [8]. While evidence suggests that any age group may have heat-health risks specific to their physiology and behaviors, there is a need for greater integration of the emerging child-focused evidence base into heat adaptation strategies. Below, we use one tool—the HVI—to illustrate how research on heat and child health could be further incorporated into existing tools and interventions.

## 3. **The** HVI

The identification of vulnerable subpopulations can inform the development and implementation of strategies to prevent heat-related morbidity and mortality. In recent years, such information has been used by both researchers and public health practitioners in order to identify spatial patterns of heat vulnerability that can be used to inform decisions about resource allocation during periods of hot weather (e.g. the placement of cooling centers). For example, the New York State Department of Health obtained census-tract level information on demographic, socioeconomic, and built environment characteristics associated with increased risk of heat-related health impacts and used principal component analysis to develop and map a composite measure of heat vulnerability to guide adaptation resource planning [9, 10]. Such composite measures have been developed for a number of communities in the United States and abroad and are typically referred to as a heat vulnerability index (HVI) [11]. While methods for developing HVIs vary, they typically involve (1) identifying characteristics that are associated with increased risk of heat-related health impacts and are thought to be relevant to the population for which the HVI is being developed; (2) obtaining fine spatial-scale data on those characteristics or closely related proxies for the population of interest; (3) creating a composite index of heat vulnerability, often using a dimension-reduction technique such as principal component analysis or factor analysis; and (4) mapping the resulting index. Finally, some studies include a validation step, in which researchers test whether the HVI is associated with an increased risk of heat-related mortality and/or morbidity on hot days in the study area.

## 4. Inclusion of child-relevant data in existing HVIs

As HVIs are intended to serve as a composite measure reflecting multiple dimensions of heat vulnerability, researchers aim to include a wide range of characteristics associated with increased heat-related health risks as inputs to the HVI development process. For example, the New York State HVI described above was constructed from 13 census tract-level variables representing vulnerability due to wide range of characteristics, including older age, social isolation, race/ethnicity, income, and land cover type [9]. Yet despite the well-documented vulnerability of children, few published HVIs include characteristics that are specifically relevant to this population. Furthermore, few such studies include health measures relevant to children in their validation stages. Specifically, many existing HVIs are validated against mortality data, yet the health impacts of heat on children—while severe and potentially long-lasting over many future decades of elevated heat—are rarely fatal [8].

To illustrate this point, here we present information that we extracted from all HVIs included in a recent systematic review by Niu and colleagues [11]. This review consisted of peer-reviewed manuscripts that described both the development and the validation of an HVI and were published between January 2010 and October 2020 in either English or Chinese. For each of the 13 studies included in the Niu *et al* review [9, 12–24], we extracted (1) a list of all variables included or considered for inclusion in the HVI and (2) a description of the health dataset(s) used to validate the HVI. We then examined the extracted information to identify whether each HVI captures heat vulnerability among children in the development and/or validation stages.

Among the 13 studies included in the Niu *et al* systematic review, all but one included at least one variable representing vulnerability based on age in the HVI (e.g. percentage of the population in a vulnerable age group within the spatial unit of the HVI). Yet while there is evidence that both older adults and children are vulnerable to heat, we found that 12 HVIs included a variable representing the percentage of the population age 65 and older while only one HVI included an equivalent variable for children (table 1). Specifically, the HVI developed for Travis County, Texas included a variable for the percentage of the population age 0–5 years old in each census block group in the study area [19]. Additionally, HVIs developed for Chicago, Illinois [12] and Greater Vancouver, British Columbia (Canada) [21] considered child-specific age group variables but did not move them forward for inclusion in the final model.

**Table 1.** Selected characteristics of studies included in a 2021 systematic review of heat vulnerability index development and validation (Niu *et al* [11]). Adapted from [11]. CC BY 4.0.

Publication	Location	Age-related variables included in HVI development	Dataset(s) used in HVI validation
Johnson *et al* [12]	Chicago, Illinois (USA)	Standardized measures of: • Females age 0–4^a^ • Males age 0–4^a^ • Population age 0–4 under the poverty threshold^a^ • Population age 25 and older with less than a high school education • Population 25 and older with a high school education • Females age 65 and older • Males age 65 and older • Females age 65 and older living alone • Males age 65 and older living alone • Population age 65 and older in group living • Population age 65 and older below the poverty threshold^a^	• Residential heat deaths (Illinois State Vital Records Department)
Reid *et al* [13]	California, Massachusetts, New Mexico, Oregon, and Washington (USA)	Percentage of population: • Age 65 and older • Age 65 and older and living alone	• Deaths due to all causes, cardiovascular disease, and respiratory disease (California Office of Vital Records, Center for Health Statistics; Massachusetts Department of Public Health Registry of Vital Records and Statistics; New Mexico Department of Health, Epidemiology and Response Division, State Center for Health Statistics; Oregon Health Authority, Public Health Division, Center for Health Statistics; Washington State Department of Health, Center for Health Statistics) • Hospital admissions due to all internal causes, cardiovascular disease, respiratory disease, cerebrovascular disease, electrolyte imbalance, heat-related illness, nephritis and nephritic syndrome, and acute renal failure (California Office of Statewide Health Planning and Development; Massachusetts Division of Healthcare, Finance, and Policy, Hospital Inpatient Discharge Database; New Mexico Health Policy Commission, Office of Health Policy and Research, New Mexico Hospital Inpatient Discharge Data; Oregon Health Authority; Washington State Department of Health, Center for Health Statistics, Comprehensive Hospitalization Abstract System)
Harlan *et al* [14]	Maricopa County, Arizona (USA)	Percentage of population: • Age 65 and older Age 65 and older and living alone	• Heat-related deaths (Maricopa County Department of Public Health surveillance system)
Wolf *et al* [15], [16]^b^	London (UK)	• Percentage of population over age 65	• All-cause deaths (UK Office of National Statistics) • All-cause ambulance call-outs (UK National Health Service)
Maier *et al* [17]	Georgia (USA)	Percentage of population: • Age 25 and older and does not hold a high school degree • Age 65 and older • Age 65 and older and living alone	• All-cause deaths (US Centers for Disease Control and Prevention, National Center for Health Statistics)
Chuang and Gober [18]	Phoenix, Arizona (USA)	Percentage of population: • Over age 65 • Over age 65 and living alone	• Hospital admissions due to heat stress (Arizona Department of Health Services hospital discharge databases)
Prudent *et al* [19]	Travis County, Texas (USA)	Percentage of population: • Age 0–5 years • Age 65 and older	• Deaths due to cardiovascular disease (Texas Department of State Health Services)
Kim *et al* 2017	Korea	Number of people: • Age 65 and older • Age 65 and older and living alone	• Heatwave-related deaths (Korean Statistical Agency)
Krstic *et al* [20]	Greater Vancouver, British Columbia (Canada)	Population density of those: • Population density, age 0–10 (⩽10)^c^ • Age 65 and older	• All-cause deaths excluding transport accidents (British Columbia Vital Statistics Agency)
Nayak *et al* [9]	New York State (USA)	Percentage of population: • Age 18–65 that has a disability • Age 18–65 that are unemployed • Over age 65 • Over age 65 and living alone	• Emergency department visits and hospitalizations for heat stress (New York State Department of Health, Statewide Planning and Research Cooperative System database)
He *et al* [22]	Shanghai (China)	None	• Heatwave deaths (Pudong Centre for Disease Control)
Mallen *et al* [23]	Dallas, Texas (USA)	Percentage of population: • Over age 65 • Over age 65 and living alone	• Heat-related deaths (US Centers for Disease Control and Prevention)
Conlon *et al* [24]	Detroit, Michigan (USA)	Percentage of population: • Over age 65 • Over age 65 and living alone	• All-cause deaths (Michigan Department of Community Health)

^a^ Variable considered for inclusion in the heat vulnerability index but excluded from the final model due to complex structure in principal component analysis loadings.

^b^ This heat vulnerability index is described across two papers by Maier and colleagues, one focused on the development and the other focused on validation.

^c^ Variable considered for inclusion in the heat vulnerability index but excluded from the final model due to lack of clear inflection point in association with mortality.

We also examined variable lists for other characteristics related to the distribution of children within a study area (i.e. the number of schools, childcare facilities, or playgrounds); however, none of the studies included this type of information. Similarly, we examined variable lists for characteristics that might indicate lack of access to cooling infrastructure specifically among children, such as the prevalence of air conditioning within schools or childcare centers. Several studies included a general measure of air conditioning prevalence in homes [13, 18, 23], but none had measures on child-specific settings, potentially due to lack of data availability, although no explanation is provided by the HVI manuscript authors.

The lack of inclusion of child-specific variables may have implications for the performance of HVIs specifically in this age group. Nayak *et al* validated their New York State HVI against both all-cause and age group-specific rates of hospital admission and emergency department visits for heat stress. For most age groups, rates of heat stress morbidity among most age groups increased monotonically with increasing HVI score, suggesting that the neighborhoods with higher HVI scores do have higher rates of a quantifiable, heat-related health outcome. However, this same pattern was not observed when examining morbidity rates among individuals age 10–17, suggesting that the HVI does not fully capture variation in the spatial pattern of vulnerability among younger individuals. However, we are unable to evaluate whether the inclusion of child-specific variables would have improved HVI performance in this age group, nor do we have information about the performance of other HVIs included in the systematic review in predicting heat-related health outcomes among children, as only the Nayak *et al*. HVI conducted a validation by age group.

The type of dataset that researchers use to validate an HVI may affect the extent to which its performance among children can be determined. While a large body of literature has documented that heat is associated with increased risk of mortality among adults [5], heat impacts among children primarily manifest as morbidity rather than mortality [8]. As such, validation performed using mortality data may not adequately measure the performance of the HVI among the very young. Of the 13 HVIs included in the Niu *et al* review, the majority (nine) were validated using mortality data only (table 1). The validation of both current and future HVIs against clinical or even preclinical datasets (e.g. hospital admission or emergency department visits, retail store sales receipts of rehydration solutions via a syndromic surveillance system), could provide further insight into their performance across the spectrum of age and could lead to the need to standardize outcomes with a health burden metric (such as disability adjusted life years (DALYs)) or develop separate HVIs to be able to highlight different health risks by age or outcome.

## 5. Summary and recommendations

The persistent health risks of extreme heat in the present day and the projected trends towards more frequent and severe heat events in the future highlight a critical need to develop, refine, and tailor strategies to protect population health during periods of hot weather. HVIs are a tool that can guide the allocation of resources during periods of extreme heat, and may also be used to inform urban planning decisions to mitigate heat exposure, such as increasing urban tree canopy cover. Yet as illustrated above, many HVIs do not include child-specific data in the development and/or validation stages despite evidence that children may be vulnerable to heat in distinct ways due to physiological and behavioral differences from adults. Further, effective protective measures may differ by age group as, for example, interventions to reduce heat-associated morbidity among pre-school aged children would likely differ from public health or clinical risk communication strategies to reduce heat-health burden among teenagers involved in outdoor work or sports.

Recommendations for how HVIs might be expanded to better capture heat-health risks among children include performing the validation step among different age groups, validating the HVI using one or more measures of morbidity in addition to mortality (e.g. by standardizing outcomes with DALYs or stratifying by outcome), developing age-group specific HVIs, and/or incorporating child-specific variables in the development process. For this latter recommendation, we acknowledge that the availability of child-relevant variables beyond simply the proportion of children living in an area may be limited. We note, however, that even the identification of such data availability limitations in the development and implementation of HVIs may have benefits. In this era of considerable investment in climate-health resilience and research, identifying where data gaps exist (e.g. widespread, high-quality data on air conditioning access in school or child care settings) has potential to catalyze new data collection efforts. Finally, while this commentary has illustrated how heat-health risks among children could be incorporated into a specific heat adaptation tool, we encourage practitioners and researchers to consider how other heat interventions—and the evidence base used to inform them—could be better tailored to protect the health of younger populations.

## Data Availability

No new data were created or analysed in this study.
